# Implementation of Multi-level Interventions to Mitigate Risk of SARS-CoV-2 Delta Variant at a PUBLIC UNIVERSITY in Southern United States

**DOI:** 10.1017/dmp.2022.179

**Published:** 2022-07-27

**Authors:** Keena N. Arbuthnot, Rebecca C. Christofferson, Edward J. Trapido, John H. Pardue, John N. Perret, William F. Tate

**Affiliations:** 1 School of Education, College of Human Sciences and Education, Louisiana State University, Baton Rouge, LA, USA; 2 Department Pathobiological Sciences, School of Veterinary Medicine, Louisiana State University, Baton Rouge, LA, USA; 3 School of Public Health, Louisiana State University Health Science Center New Orleans, New Orleans, LA, USA; 4 Department of Civil & Environmental Engineering, College of Engineering, Louisiana State University, Baton Rouge, LA, USA; 5 LSU Student Health Center, Division of Student Affairs, Louisiana State University, Baton Rouge, LA, USA; 6 Department of Sociology, College of Humanities and Social Science, Louisiana State University, Baton Rouge, LA, USA

**Keywords:** COVID-19, SARS-CoV-2, pandemic response, higher education, public health

## Abstract

During the coronavirus disease 2019 (COVID-19) pandemic, navigating the implementation of public health measures in a politically charged environment for a large state entity was challenging. However, Louisiana State University (LSU) leadership developed and deployed an effective, multi-layered mitigation plan and successfully opened in-person learning while managing cases of severe acute respiratory syndrome coronavirus 2 (SARS-CoV-2) during the fourth surge. We describe the plan to provide a framework for other institutions during this and future responses. The goals were 3-fold: maintain a quality learning environment, mitigate risk to the campus community, and ensure that LSU operations did not contribute to health-care stress. As of September 2022, LSU has achieved high compliance with interventions and relatively low virus activity on campus compared with peer institutions. This university model can serve as a template for similar implementation plans in the context of complex socio-political and economic considerations.

This essay describes the efforts to create a sustainable community of teaching and learning during the fourth surge of the severe acute respiratory syndrome coronavirus 2 (SARS-CoV-2) pandemic in Louisiana, an effort encapsulated in the realities of the politics of the American South yet informed by the lessons of public health and epidemiology. The modern research university, populated by scientists, is a primary promoter of science, and represents one of society’s pillars of discovery and translation. However, the public flagship university operates in a political ecosystem, where science and politics co-exist and at times are at odds. For instance, Louisiana law prohibits a true universal vaccine mandate for students enrolled in public universities, as post-secondary institutions must accept blanket exemptions for religious or personal reasons. In addition, in May 2021, the state’s attorney general urged LSU not to pursue a vaccine mandate for students and, in July 2021, threatened to sue a private university if they implemented such a mandate.^1^ At the same time, Louisiana began to see a drastic increase in the number of cases of the Delta variant of SARS-CoV-2.^2^ It became evident that Louisiana and East Baton Rouge Parish were trending toward a fourth surge.^3^ Thus, the combination of regulatory and political tensions coupled with current and expected continued increase in transmission intensity presented design challenges for the operation of the Louisiana State University Agricultural & Mechanical College (LSU) campus.

In the months prior, Louisiana had experienced a relative lull in the pandemic.^4^ The state had moved away from restrictions on the capacity of businesses, lifted the mask mandate, and although vaccination rates were low, the incidence of both cases and hospitalizations remained manageable. However, in late June 2021, the first case of the Delta “variant of concern” was identified in New Orleans.^5^ By the end of July, the majority of cases in Louisiana were Delta.^6^ During that time, it was evident that this surge—driven by a low vaccination rate in the state, the Delta variant, and a lack of social restrictions, and nonpharmaceutical interventions— caused major stress on health-care systems. By July 27, 2021, there had been a 225% increase in cases across the state compared with the first week of July. Louisiana had one of the lowest vaccination rates in the nation, with only 37% of the state reported being vaccinated by August 10, 2021. At this same time, Louisiana had the highest rate of coronavirus disease 2019 (COVID-19) cases in the nation, second only to Fiji in the world in this category.^7,8^

Louisiana hospitals, including the largest hospital in the state, located in Baton Rouge, were running out of intensive care unit (ICU) beds, having been filled almost exclusively by unvaccinated COVID-19 cases.^9^ In mid-August, over 88% of staffed ICU beds in the state were occupied, and almost 75% of total staffed hospital beds were occupied.^10^ In East Baton Rouge Parish, COVID-19 patients accounted for 62% occupancy of staffed ICU beds and almost 33% for total staffed hospital beds (as of August 18, 2021).^11^

At the point LSU began preparing for the start of the Fall 2021 semester, cases in East Baton Rouge parish, the location of the main LSU campus, constituted 9.4% of the state’s total positive cases.^12^ According to Louisiana Governor John Bel Edwards, “The White House has notified Louisiana that we are a State of Concern because we are the leading edge of the COVID-19 surge… Indeed, Louisiana leads the nation in case of growth… This should come as no surprise to anyone who has watched our case counts and hospitalizations continue to climb.”^13^

During this period, LSU leadership was tasked with pivoting from the expected “return to normal” Fall 2021 semester based on interpretations of late spring-early summer data to dealing with another surge of the pandemic. With LSU classes beginning on August 23, preceded by orientation in the week(s) before, over 30,000 students were potentially returning within the peak incidence period. The President and leadership team developed Return to Campus policies that ensured a safe return to campus for all LSU students, faculty, and staff.

The challenges of developing an effective and comprehensive Return to Campus plan were exacerbated by an apparently low vaccination rate among students, a requirement to live on-campus for most incoming freshmen, and a politically charged environment that stymied public health interventions. Furthermore, pandemic fatigue had set in after a year of shutdowns and constant polemic debate, much based on misinformation and/or misrepresentation of data.

While successful in the preceding year, these new sets of circumstances (ie, fourth surge, completely open state, Delta variant, and the imminent start of school) required the design and implementation of an updated, comprehensive COVID Response Policy Model built on what had been learned from scientific advancements and campus experience. At the time the LSU leadership team began formulating policies, 3 major contexts became priorities over a matter of days. First, LSU needed to pivot its plan from the previous years to deliver face-to-face instruction to students in the context of a state that was not shut down but in the midst of intense SARS-CoV-2 transmission. Second, LSU needed to ensure that, with all of the tools and data available, it was operating in a manner to mitigate the risk to community members to the best of its ability. Finally, LSU needed to ensure that it did not, as a result of operations, contribute to the increasing health-care stress of the surrounding communities.

## Methods

### LSU COVID Response Policy Model

**Figure 1.**
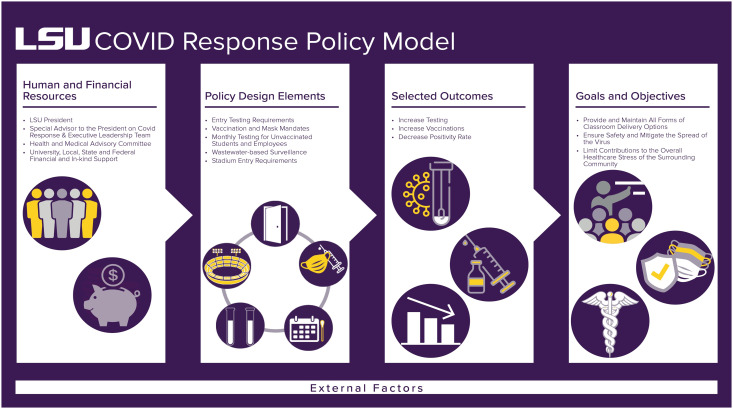
LSU COVID Response Policy Model.

The LSU COVID Response Policy Model is a broad evaluative framework that accounts for a multitude of internal and external factors. Specifically, the LSU leadership team developed the model to enable continuous assessment of the effectiveness of the LSU COVID Response Policy. Through systematic application of the framework and proper implementation of individual design elements, the LSU leadership team had the flexibility and versatility to swiftly pivot and make policy and programmatic improvements. This model takes into consideration the (a) availability of human and financial resources, (b) policy design elements, (c) expected outcomes associated with the policy implementation, and (d) overarching goals and objectives. In addition, this framework provides for an extensive examination of the external factors that could impact the success of the proposed model (Figure 1).

### Goals and Objectives

The goals and objectives of the LSU COVID Response Policy Model are 3-fold. First, LSU needed to be in a position to continue to support the Vision and Mission of the university by maintaining learning environments with all modes of delivery (ie, face-to-face, online, hybrid). Consistent with a majority of the higher education institutions across the country, LSU needed to provide most of its courses face-to-face in the Fall of 2021. Second, LSU needed to mitigate the spread of the virus to ensure the health and safety of the campus community. Finally, LSU needed to ensure that it not contribute to the increasing health-care stress of the surrounding communities as a result of operations. Consequently, the leadership team built the LSU COVID Response Policy Model to reach these overarching goals and objectives.

### Human and Financial Resources

The LSU COVID Response Policy Model capitalized on alignment between leadership in the University and System President’s Office and the executive leadership team, which included a special advisor to the President whose duties focused on the COVID Response Policy design and implementation. In addition, the leadership team appointed a Health and Medical Advisory Committee consisting of faculty and community practitioners with expertise in epidemiology, clinical diagnosis, environmental science, statistics, psychometrics, and virology to provide scientific support and guidance on the policy design and implementation process. The LSU COVID Response Policy Model uses federal, state, local, and university financial (ie, CARES Act funding) and in-kind resources (ie, testing assets). The commitment of the necessary human and financial resources proved to be critical to the success of the COVID Response Policy Model, as groups met multiple times per week to communicate changes in the state of the pandemic, the community, and the university.

### LSU COVID Response Policy Design Features

The overarching goal of the LSU COVID Response Policy Model was to ensure that it continued to meet the vision and mission of the university while enabling the university to continue to offer (a) face-to-face classroom experiences, (b) mitigating the spread of the virus, (c) vaccinating a majority of the campus, and (d) not contributing to the stress and strain of the local and surrounding health-care community. The model used a multi-faceted approach to provide actionable intelligence useful in reaching the stated goals and objectives. The policy design consists of 5 interdependent pillars, (i) entry testing requirements, (ii) vaccine and mask mandates, (iii) wastewater-based surveillance and response, (iv) testing regimen for unvaccinated students and employees, and (v) football stadium entry requirements. In addition, we instituted compliance measures to ensure the maximum impact of each design feature. These measures range from remediation through either student affairs or human resources to dismissal from the university. In addition, the model seeks to inform the development of a sustainable community of teaching and learning in the context of the pandemic.

## Entry Requirements

University leadership provided the students the option to produce 1 of 3 verifications to gain entry to campus in Fall 2021. These options included students providing documentation of either a COVID-19 vaccination, proof of a negative COVID-19 test result no more than 5 d before campus arrival, or a positive COVID-19 test no more than 90 d before campus arrival. With a rapid deteriorating situation in the regional and state communities and health-care systems stressed to capacity, the goal was to “catch and interrupt” potential transmission chains of the highly infectious Delta variant upon opening campus.^14,15^ We deemed this of critical importance for residential students moving into congregate settings. In addition, we used entry testing to test residential students returning to campus after Hurricane Ida, given the likely unavoidable disruption of nonpharmaceutical interventions.

Leadership made specific determination of element parameters with both science, social, and logistical considerations. For example, out-of-state students who were positive would need to stay home. This 5-d period for testing would allow for the receipt of the test and appropriate planning around travel should the student be positive. Additionally, at that time, the upper limit of the turnaround time for polymerase chain reaction (PCR) tests offered on campus, as well as the freely available testing provided by the Louisiana Department of Health (LDH), was 5 d. The incubation period of the Delta variant was estimated to be 4 d (compared with 6 d of other variants), so this 5-d window was acceptable given the other considerations.^16^

## Vaccine and Mask Mandate

Early in the pandemic, LSU instituted a mask mandate. Louisiana continues to occupy a unique place in the pandemic, with our first and fourth surges coming sooner than a large proportion of the country. Thus, the often more mature experiential evidence in our community led to more conservative policies than peers, including not only the scientific evidence but the socio-political context in which that evidence could be presented and implemented. For example, in Spring 2020, the Centers for Disease Control and Prevention (CDC) altered its guidance on masking, indicating that unvaccinated individuals could go unmasked.^17^ However, at that time, breakthrough infections had begun appearing in Louisiana, and LSU made the decision to keep the mask mandate in place on campus due to 2 factors (1) the increasing occurrence of breakthrough infections and (2) the understanding that removal of a mask mandate would mean harder reinstatement should it become necessary. The latter proved true when, in August 2021, Louisiana Governor John Bel Edwards reimposed an indoor mask mandate that met with much social chagrin and less observable compliance. Unsurprisingly, as transmission intensity continued to increase in the state and the communities surrounding LSU, breakthrough cases also increased, making masking critical to control the spread of SARS-CoV-2.^18^

Given the severity of the fourth surge in Louisiana, vaccinating students, faculty, and staff became a necessary policy decision to protect the LSU community from the possibility of severe disease and/or hospitalization. The LSU President announced an intent to mandate a SARS-CoV-2 vaccine immediately following full United States Food and Drug Administration (FDA) approval on August 3, 2021, which came August 23, 2021. The LSU President announced the vaccination mandate the day after the FDA’s full approval. The mandate included state-allowed exemptions as required by Louisiana law. Furthermore, by minimizing the potential for severely ill individuals from the LSU community, we avoided additional stressors on the health-care system. Understanding the ecosystem of available vaccines globally, we determined [or established] a set of conditions that met the requirement for vaccination in place of receiving the mandated Pfizer vaccine. This included acceptance of other FDA emergency use authorized vaccine candidates (Moderna, Janssen) as well as internationally approved vaccines (eg, Astra-Zeneca.^9,20^

LSU is part of the Southeastern Conference (SEC), which encompasses 14 peer institutions. Only 50% of schools in the SEC reported vaccination rates publicly as of the beginning of the Fall 2021 semester. LSU was 1 of 3 schools who reported data with a vaccination rate over 80%. Other schools reported rates of between 58% and 69%. One school reported an approximate number of vaccinated individuals which resulted in an estimation of at most 32.3%. Additionally in the state of Louisiana, LSU led in vaccination rates at nonmedical higher education institutions, where other entities who reported did so at rates between 40% and 68%.

### Wastewater-Based Surveillance and Response

Testing campus wastewater for SARS CoV-2 allows anonymous surveillance of populations in residential settings on LSU’s campus.^21^ LSU’s wastewater surveillance program, active since the Fall of 2020, currently monitors 26 locations weekly, covering all dormitories, Greek houses, and on-campus apartments where students reside. A faculty member in the LSU College of Engineering heads the wastewater surveillance team. Additionally, the team monitors 2 professional schools (Law School and Veterinary Medicine), and on-campus auxiliary facilities (K-12 school and preschool) with vulnerable populations. Sampling locations can be associated with multiple dorms, campus apartment complexes, or Greek houses, typically targeting 250-500 students per location. The goal of the program is to identify when >2% of the residents in a dormitory are infected and actively shedding the virus, equivalent to 5-10 residents in most sampling locations. The testing strategy consists of the collection of composite time-weighted wastewater samples early in the day, biased toward periods of increased shedding from the residential campus community. The team compares SARS CoV-2 virus concentrations with a predetermined trigger (90,000 genome units/L), determined from previous monitoring of wastewater collected from large treatment plants in East Baton Rouge Parish. The selected trigger value, a basin-wide average measured during Louisiana’s 2^nd^ COVID surge (June/July of 2020), represents a viral concentration associated with high levels of community spread and disease incidence. Even though determined from 24-h flow-weighted composites from large wastewater plants, the value represents an appropriately conservative comparative value given the biased samples collected during campus sampling and the differences in water use profiles between dorm residents and individual homes. Once triggered, medical teams test dorms immediately, usually moving door to door within the dormitory, and quarantining or isolating impacted students, as appropriate.

The wastewater detection program has been effective at identifying locations of increased disease incidence, driving individual testing in dormitories where the trigger value was exceeded. The wastewater testing program also proves effective at tracking overall viral concentrations in residence hall wastewater, which can then be compared with the incidence in city wastewater, representative of viral loads in the broader Baton Rouge community.

### Testing Regimen for Unvaccinated Students and Employees

We surmise that unvaccinated individuals still account for the majority of transmission, where the CDC estimates that unvaccinated individuals are 5 times more likely to be diagnosed with SARS-CoV-2 compared with vaccinated individuals.^22^ Thus, LSU requires all unvaccinated students and employees to get tested once a month, and just over 6000 individuals are currently in the mandated testing program. Every week of each month, the university randomly selects and requires 25% of unvaccinated students and employees to get tested, with students stratified based on residence status (on- vs off-campus). While imperfect, this model had 2 major benefits. First, rather than be tested, a subset of unvaccinated individuals chose to begin the vaccination process. Second, weekly data provided incidence data and positivity rates to deduce transmission trajectories, in addition to identifying individuals who might need medical follow-up. At the end of August 2021, Hurricane Ida interrupted this process, after which return-to-campus testing was required for many residential students. Thus, we adjusted the model to account for those individuals that had tested upon return to campus, illustrating the interconnectedness of the design elements and flexibility of the overall model.

### Large Stadium Entry Requirements

LSU Tiger Stadium holds 102,321 persons but tailgating brings in an estimated 75,000+ additional people to campus who watch the game on LSU grounds. This environment concerned us as it was ripe for super spreader events, especially if it went forward with limited mask-wearing, inadequate social distancing, and the low rate of vaccination in the state at that time. Central to the idea that LSU does not contribute to the stress on health-care and the overall spread of the virus was the LSU leadership team’s strong conviction that outdoor athletic events align themselves with the goals of the LSU COVID Response Policy Model. The policy model requires attendees to show proof of vaccination or negative PCR test within 72 h upon entry. Moreover, the university made additional antigen testing available at the stadium on the day of the game. The model encourages children under the age of 5 to mask, and children 5-11 were asked to mask. While the implementation of these interventions poses its own set of challenges, the impact of these events on the pandemic continues to be monitored and plans adjusted. In addition, given the idea that such programs associated with the university could be used to improve not only the public health on the campus in conjunction with its hospital partners, but LSU also gave unvaccinated individuals the convenient opportunity to get their first vaccine dose.

### LSU COVID Data Ecosystem

The 5 policy design elements of the LSU COVID Response Policy Model are embedded within a well-established data ecosystem. The leadership team designed the LSU COVID data ecosystem to collect data regularly from a myriad of sources to monitor the situation within the LSU community, the East Baton Rouge Metropolitan area, and the state of Louisiana. Data-driven decision-making represents 1 of the most critical components of LSU’s COVID Response Model, with data integration structures developed to facilitate data analysis for the various reports. For example, the LSU leadership team gathered and reported community metrics to gauge stress on the health-care system multiple times weekly, measured updated case counts in near real-time, and dashboards for administrators provided up-to-date information about COVID on and off-campus (ie, positive cases, quarantine, isolation, hospital and ICU bed usage in the region and state). This required data integration across campus testing entities and sites, as well as with the daily symptom checker and self-reporting platform. LSU leadership team examined active cases, as well as cumulative case numbers and trends, with respect to classrooms, organizations (including Greek and athletics), and residence halls for possible clusters (defined as 2 or more currently active cases). Within this data ecosystem, the dynamics of COVID on campus could be monitored by LSU administrators in near real-time.

## Results

### Entry Requirements

Over 99% of the students complied with the entry requirements, providing either proof of vaccination, negative test, or positive test within 90 d. Over 7000 residential life students tested, provided proof of vaccination or positive test before campus entry, with 50 positive cases detected and required to isolate at home before campus entry. Furthermore, upon returning to campus after Hurricane Ida, residence halls occupants required to test again resulted in 23 positives who were isolated immediately. With a basic reproductive number estimated to be greater than 5,^15,23^ these 73 cases represent a significant interruption of potential chains of transmission, especially in a congregate setting.

### Vaccine and Mask Mandates

Between the President’s announcement that a mandate would be forthcoming (August 3, 2021) and the issuance of said mandate (August 24, 2021), the percent of students who reported vaccination status increased, and the student vaccination (at least 1 shot) rate rose from 63% to 77.8%. Since the implementation of the mandate, the percent of students at least partially vaccinated has increased to 82.3%. During the interval from the initial announcement to the mandate, there was a 14.8% increase in vaccination compliance (reporting or initiation) compared with 4.5% increase since the time of the mandate. This demonstrates the potential benefit of an intent-to-mandate announcement in conjunction with required entry testing in kick-starting increased compliance.

### Wastewater-Based Surveillance and Response

During the 2020-21 academic year, sampling locations triggered 7 times, resulting in testing in 11 residence halls. To date, in the 2021-22 academic year, sampling locations have triggered 4 additional times resulting in the testing of 6 dorms. If wastewater results indicate the presence of virus, the LSU Covid Response team sends a crew of university and vendor personnel to the location to test all residents regardless of vaccination status. In 3 of the 4 locations in the 2021-22 academic year, positive students were identified consistent with program goals. In the 2020-21 academic year, 5 of the 7 sampling locations yielded effective results. To date, the program has been effective in identifying 49 students in the 2021-22 academic year, many asymptomatic, potentially breaking transmission chains. Occasionally, patterns of positive detections also revealed clusters of positive students consistent with the transmission in the dorm. In every case, the following week’s testing revealed viral concentrations well below the trigger once Residential Life separated impacted students from the population at-large.

### Testing Regimen for Unvaccinated Students and Employees

During the first several weeks of testing, beginning the week of September 20, the results showed that the positivity rate for unvaccinated students was 0%, 0.84%, and 0.22%, respectively. These data, in conjunction with other data sources (ie, positivity rate from all on-campus testing sites, positivity rate of the surrounding community), confirmed further that the virus spread on campus continued to improve. The results from the monthly testing program will continue to help the LSU administrators monitor the virus spread on campus, especially in the unvaccinated population.

### Large Stadium Entry Requirements

Preliminary data from wastewater testing on campus and in the surrounding community suggests that the first 2 home football games did not markedly increase the prevalence of the virus on campus, nor has there been a significant increase in positive cases in the LSU community. In fact, the weeks after the first 2 home games had the lowest amount of virus in the wastewater samples compared with previous weeks. The number of cases and hospitalizations state-wide continued to decrease in the days following these games, and the positivity rate on campus continued its downward trend, which indicates that hosting these games with these mitigation measures met the objective of not contributing to state and regional health-care stress and overall transmission. Furthermore, at the first home game, over 100 vaccinations were initiated. LSU Athletics lifted the requirements for entry at the stadium over a month into the semester once the infection rate at the university and the surrounding community were sufficiently lower. The LSU leadership team continues to review and will report the outcomes associated with these modifications.

## Discussion

The LSU fight against COVID was recently noted as an early adopter and rated exemplar by the White House.^24^ The response has been comprehensive, responsive, and flexible to meet the unique needs of our community. It included the components of the CDC and LDH recommendations, with a transdisciplinary coordinating team implementing and monitoring its effectiveness. All the policy design elements contributed to the ongoing success of the LSU COVID Response Policy Model. While it is tempting to declare victory, science and experience caution us otherwise.

The landscape in which the LSU leadership team constructed this response included a politically and socially polarized climate, external pressures of contradicting politics, and the 2-pronged effects of misinformation leading to unwillingness or hesitancy of pharmaceutical mitigations. This necessitated a delicate balance of scientific, logistical/financial, and social factors integrated into the response plan, as an unbalanced approach could have led to the significant financial instability of the university, leading to long-term deleterious effects on higher education at the state’s flagship campus. The LSU COVID Policy Model has managed to maintain a semblance of balance whereby we have successfully opened the academic year amid the fourth surge of a new, more transmissible variant of SARS-CoV-2.

## Conclusions

In summary, LSU—a relatively large organization with diverse cultures and beliefs and often under contrasting political pressures—has been successful in developing a plan that addressed the multifactorial needs of a large university and its surrounding community. LSU has one of the most conservative approaches to COVID-19 mitigation among peer institutions, which was in part instituted successfully due to aligned university leadership and support from governmental officials. Furthermore, by using a multi-sectoral approach combining organizational operations, science, and public health, LSU has managed to return to campus and resume new-normal operations. The leadership team continues to monitor the situation both within and outside the LSU community, including new rises in cases in parts of the country where no fourth major surge has yet occurred. The LSU leadership team hopes to offer this model as a framework upon which similar multidisciplinary plans can be built that account for university and community health in the context of complex socio-political and economic considerations.

## References

[1] Fox 8 Staff. Mandating COVID vaccinations at LSU is illegal, says La. Attorney General. 2021. Accessed July 31, 2022. https://www.wafb.com/2021/06/01/mandating-covid-vaccinations-at-lsu-is-illegal-says-la-attorney-general/

[2] Bruhl A. Louisiana Department of Health confirms delta variant in the state. KLFY.com. 2021. Accessed October 29, 2021. https://www.klfy.com/health/coronavirus/louisiana-department-of-health-confirms-delta-variant-in-the-state/

[3] Weiss B. About 100 new patients hospitalized with COVID-19: delta variant increasing case numbers. 2021. Accessed August 1, 2022. https://www.wbrz.com/news/about-100-new-patients-hospitalized-with-covid-19-delta-variant-increasing-case-numbers/

[4] New York Times. Tracking coronavirus in Louisiana: latest map and case count. 2021. Accessed August 1, 2022. https://www.nytimes.com/interactive/2021/us/louisiana-covid-cases.html

[5] Westwood R. ‘This should scare the hell out of you’: New Orleans officials warn delta variant is here and ‘killing people’. 2021. Accessed August 1, 2022. https://www.wwno.org/public-health/2021-07-13/this-should-scare-the-hell-out-of-you-new-orleans-officials-warn-delta-variant-is-here-and-killing-people

[6] Westwood R. Delta is now the dominant coronavirus variant in Louisiana, cases on the rise. 2021. Accessed August 1, 2022. https://www.wwno.org/public-health/2021-07-08/delta-is-now-the-dominant-coronavirus-variant-in-louisiana-cases-surge

[7] Michelson A. Louisiana has a higher rate of COVID-19 infections than every country but one. 2021. Accessed August 1, 2022. https://www.businessinsider.com/louisiana-has-more-covid-cases-per-capita-than-any-country-2021-8

[8] Potter WT , Stucka M. Louisiana: the rare case of a state ravaged twice by COVID-19. 2021. Accessed August 1, 2022. https://www.usatoday.com/in-depth/news/2020/08/01/louisiana-second-covid-19-wave-worse-than-first-no-1-per-capita/5558862002/

[9] Adelson J , Woodruff E , Myers B. Louisiana hospitals are under strain as coronavirus cases surge: ‘Who can come to help?’. 2021. Accessed August 1, 2022. https://www.nola.com/news/coronavirus/article_4fa0e548-c87d-11ea-ba0a-77bf2cce6d45.html

[10] The Louisiana Department of Health. COVID-19 information. 2021. Accessed August 1, 2022. https://ldh.la.gov/coronaviru, 2021.

[11] CDC. COVID data tracker. Accessed August 18, 2021. https://covid.cdc.gov/covid-data-tracker/#county-view?list_select_state=all_states&list_select_county=22033&data-type=Cases

[12] USA Facts. East Baton Rouge Parish, Louisiana coronavirus cases and deaths. 2021. Accessed August 1, 2022. https://usafacts.org/visualizations/coronavirus-covid-19-spread-map/state/louisiana/county/east-baton-rouge-parish

[13] Louisiana Department of Health Staff Report. New mask guidance from Governor Edwards and LDH. 2021. Accessed August 1, 2022. https://www.kedm.org/post/new-mask-guidance-governor-edwards-and-ldh#stream/0

[14] CDC. Delta variant: what we know about the science. 2021. https://www.cdc.gov/coronavirus/2019-ncov/variants/delta-variant.html

[15] Del Rio C , Malani PN , Omer SB. Confronting the Delta Variant of SARS-CoV-2, Summer 2021. JAMA. 2021;326(11):1001-1002. 34406361 10.1001/jama.2021.14811

[16] Li B , Deng A , Li K , et al. Viral infection and transmission in a large well-traced outbreak caused by the Delta SARS-CoV-2 variant. *MedRxiv.* 2021. doi:10.1101/2021.07.07.21260122 10.1038/s41467-022-28089-y PMC8786931 35075154

[17] Rodriguez A. CDC lifts indoor mask guidelines for fully vaccinated people. What does it actually mean? 2021. Accessed August 1, 2022. https://www.usatoday.com/story/news/health/2021/05/13/cdc-mask-guidelines-im-vaccinated-what-can-do-indoor-and-outdoor/5078193001/

[18] Schmaltz T. After months of inquirities from WBRZ, state releases breakthrough case percentages for vaccines. 2021. Accessed August 1, 2022. https://www.wbrz.com/news/after-months-of-inquiries-from-wbrz-state-releases-breakthrough-case-percentages-for-vaccines/

[19] World Health Organization (WHO). Status of COVID-19 Vaccines within WHO EUL/PQ evaluation process. 2021. Accessed August 1, 2022. https://extranet.who.int/pqweb/sites/default/files/documents/Status_COVID_VAX_20Oct2021.pdf

[20] World Health Organization (WHO). 11 vaccines granted emergency use listing (EUL) by WHO. Accessed August 9, 2022. https://covid19.trackvaccines.org/agency/who/

[21] Walke HT , Honein MA , Redfield RR. Preventing and responding to COVID-19 on college campuses. JAMA. 2020;324(17):1727-1728. 32991681 10.1001/jama.2020.20027 PMC9648565

[22] Bozio CH , Grannis SJ , Naleway AL , et al. Laboratory-confirmed COVID-19 among adults hospitalized with COVID-19–like illness with infection-induced or mRNA vaccine-induced SARS-CoV-2 immunity - nine states, January-September 2021. MMWR Morb Mortal Wkly Rep. 2021;70(44):1539-1544. 34735425 10.15585/mmwr.mm7044e1 PMC8568091

[23] Liu Y , Rocklöv J. The reproductive number of the Delta variant of SARS-CoV-2 is far higher compared to the ancestral SARS-CoV-2 virus. J Travel Med. 2021;28(7):taab124. 34369565 10.1093/jtm/taab124 PMC8436367

[24] Ballard M. ‘99.999%’ of LSU population compliant with COVID rules, William Tate tells Joe Biden. The Advocate. September 15, 2021. Accessed August 1, 2022. https://www.theadvocate.com/baton_rouge/news/education/article_22e34534-162c-11ec-82e9-e7b3e10984dc.html

